# Vocal Expression of Affective States in Spontaneous Laughter reveals the Bright and the Dark Side of Laughter

**DOI:** 10.1038/s41598-022-09416-1

**Published:** 2022-04-04

**Authors:** Diana P. Szameitat, André J. Szameitat, Dirk Wildgruber

**Affiliations:** 1 Department of Psychiatry and Psychotherapy, University of Tübingen, Calwerstraße 14, 72076 Tübingen, Germany; 2 Division of Psychology, Brunel University London, Uxbridge, UK; 3 Centre for Cognitive Neuroscience, Division of Psychology, Department of Life Sciences College of Health, Medicine and Life Sciences, Brunel University London, Kingston Lane, Uxbridge, UB8 3PH UK

**Keywords:** Psychology, Human behaviour

## Abstract

It has been shown that the acoustical signal of posed laughter can convey affective information to the listener. However, because posed and spontaneous laughter differ in a number of significant aspects, it is unclear whether affective communication generalises to spontaneous laughter. To answer this question, we created a stimulus set of 381 spontaneous laughter audio recordings, produced by 51 different speakers, resembling different types of laughter. In Experiment 1, 159 participants were presented with these audio recordings without any further information about the situational context of the speakers and asked to classify the laughter sounds. Results showed that joyful, tickling, and schadenfreude laughter could be classified significantly above chance level. In Experiment 2, 209 participants were presented with a subset of 121 laughter recordings correctly classified in Experiment 1 and asked to rate the laughter according to four emotional dimensions, i.e., arousal, dominance, sender’s valence, and receiver-directed valence. Results showed that laughter types differed significantly in their ratings on all dimensions. Joyful laughter and tickling laughter both showed a positive sender’s valence and receiver-directed valence, whereby tickling laughter had a particularly high arousal. Schadenfreude had a negative receiver-directed valence and a high dominance, thus providing empirical evidence for the existence of a dark side in spontaneous laughter. The present results suggest that with the evolution of human social communication laughter diversified from the former play signal of non-human primates to a much more fine-grained signal that can serve a multitude of social functions in order to regulate group structure and hierarchy.

## Introduction

Laughter is an important signal in social interaction^1^, sharing common evolutionary roots with our nonhuman primate ancestors^2^. In humans and nonhuman primates alike, laughter can serve as a playsignal^3^, promoting positive feedback and the prolongation of play^4^. However, compared to nonhuman primates, humans show more complex social interactions and, as a consequence, laughter is no longer restricted to the context of tickling and physical play. Instead, humans laugh in various affective states^5–7^. Generally, laughter has been described as an expression of positive affective states, such as amusement, joy, affection, cheerfulness, and surprise^8–10^, and is uttered in situations of social interaction^11,12^, as a response to another’s laughter^13^, or the sharing of humour^14^. In such situations, it may function to establish social bonds and strengthens group cohesion^7,15–17^. In addition to this bright side of laughter, various researchers suggested that laughter can also take on a dark side, for example when affective states of schadenfreude, taunt or triumph are expressed^7,16,18,19^. In such situations, it may function to express hierarchy or to exclude people from social groups^16^. The fact that laughter can be based on different affective states is widely accepted. However, it is still a matter of debate whether spontaneous laughter based on different affective states can be differentiated by listeners solely based on the acoustical signal independent of the knowledge about the sender’s affective state or situational context.

Previously, we have shown that laughter produced by professional actors can indeed be categorised by naïve listeners according to the underlying affective content. In addition, it was possible to differentiate the laughter based on participants’ ratings on multiple emotional dimensions, and with respect to its acoustical parameters^7,20^. Further supporting evidence has been shown by Wood et al.^21^, who examined posed laughter from a commercial sound database and found that acoustic variations in the laughter signal are linked to the perception of reward, affiliation, and dominance.

However, it is still a matter of debate whether posed laughter, which is produced on command (i.e., acted, faked, volitional laughter), and spontaneous laughter that is spontaneously emitted (i.e., genuine, authentic laughter)^22^ are comparable. Generally, it has been argued that posed expressions might not be representative of spontaneously emitted expressions in daily life^23^. In particular, affective information may be over-emphasised to fit the expected cultural norms and stereotypes, and decoding accuracy might be inflated^24^. These suggestions are supported by observed differences between posed and spontaneous laughter with respect to listeners’ perception, acoustics, and neural processing^22,25^. For example, listeners are able to tell spontaneous and posed laughter apart^22,26^. Furthermore, it has been shown that listening to spontaneous and posed laughter triggers different facial expressions^27^ and activation of different neural pathways^25,28^. With respect to acoustic properties, spontaneous laughter is longer, higher pitched, and has different spectral characteristics as compared to posed laughter^22^. Therefore, it is open whether the findings on affective communication can be generalized from portrayed to spontaneous laughter.

However, a number of findings suggest that also spontaneous laughter is capable of carrying more fine-grained information. For example, spontaneous laughter varies acoustically with respect to the social context in which it has been emitted. In detail, receivers are able to differentiate between spontaneous laughter produced in social interactions with friends versus strangers^29^, and they can derive information about social hierarchy^30^. These findings are in line with research showing that nonverbal vocalizations in general are a potent tool to communicate the sender’s affective state, such as different positive emotions^10^. Wood^31^ found evidence that differences in acoustic parameters are related to differences in the affective context of laughter, i.e., laughter emitted during the perception of videoclips evoking reward (humorous laughter), affiliation (humorous and tenderness laughter), and dominance (derisive and ridiculing laughter) showed differences in acoustic parameters. Similarly, Tanaka & Campbell^32^ have shown that in spontaneous conversations polite-formal laughs differ acoustically from mirthful laughs. However, in the current context the latter two studies leave an important question open, namely whether listeners perceive and use these acoustic differences to infer the affective state of the sender at all.

Taken together, it is still unclear whether previous findings based on posed laughter can be generalised to spontaneous laughter. Thus, the present study aimed to investigate whether the affective state of the sender can be inferred solely based on the acoustical signal of spontaneous laughter independent of the knowledge about the situational context in which the laughter has been emitted.

In the current study, groups of friends engaged in various playful activities aimed at eliciting laughter in variable situational contexts while their laughter was recorded. Affective experience for the activities was determined by a post-hoc questionnaire individually for each participant and activity. In Experiment 1, the acoustic laughter sequences were presented to naïve participants (i.e., receivers) without any further information about the situational context and receivers were asked to classify the laughter into distinct categories, such as joy, tickling, and schadenfreude in a categorial approach. If receivers are able to classify laughter sounds according to the self-reported affective state of the sender above chance level, this would be a strong indication that differences in the affective state of the sender are communicated via the acoustical laughter signal.

## Experiment 1

### Methods 1

#### Participants

In order to record spontaneous laughter, groups of 2–4 native German friends participated in various activities (N = 76 participants, 40 females, 36 males, same-sex groups: two groups of two participants each, 16 groups of three participants each, 5 groups of four participants each; mixed-sex groups: two groups of two participants each). Prior to the experiment participants received written instruction and gave written informed consent. All studies in this manuscript were approved by the ethical review board of the Medical Faculty of the University of Tübingen, Germany (225/2010BO1). All studies were carried out in accordance with the Declaration of Helsinki. Participants were left naïve to the true purpose of the experiment, i.e., they were not aware that the purpose of the study was to investigate laughter. Instead, they were told a cover up story that the study investigates the influence of humour on speech. They were encouraged to have fun and to talk and laugh freely at any time throughout the session. Sessions lasted 1.5–2 h and participants received a financial compensation of 10 Euro/hour.

#### Procedure

We used five activities to initiate fun interactions between participants, always presented in the following order: (1) reading tongue twisters; (2) watching funny video clips (set 1): (3) “animal karaoke”, where a karaoke song had to be performed in an animal voice; (4) watching another set of funny video clips (set 2), and (5) tickling. (Data for tongue twisters and “animal karaoke” are not reported here).

For watching funny video clips, participants were presented with two longer videos, lasting 14 min and 16:17 min, respectively. Each video consisted of a collection of various video clips taken from YouTube or other public resources, e.g., from the German version of “Family Feud” or “You’ve been framed”. In order to avoid serial effects, the video clips within one set were presented in different orders. The first set of video clips consisted of funny and joyful clips, such as babies laughing along with their mother. In the second set of video clips the clips aimed at the darker side of laughter. For example, they included mishaps, like people falling off bikes, and pranks, like a person stepping into mouse traps, aimed at evoking more taunting or schadenfreude laughter. Each participant used headphones and sat in front of a different screen, separated by dividing panels made from mineral wool. While participants could not see each other, they all sat at the same table and could easily talk to each other. However, most laughs were emitted in response to the video clips and not as part of a conversation among the friends.

For the tickling activity, one person sat on a chair (the ticklee) while the other participants (the ticklers) stood behind the ticklee and started tickling, usually under the arms, at the side of the chest and in the neck area. Possibly because participant groups consisted of friends and participants felt quite comfortable, this mostly worked very well except for a few participants who were not ticklish. Tickling was done in bouts repeated 4–5 times for each ticklee. Between these tickling bursts the ticklee had to read a sentence presented on the laptop (as part of the cover story). The ticklers were instructed to be as quiet as possible and not speak and laugh while tickling. However, they still often laughed or talked.

Regarding the level of authenticity, it is noteworthy that participants were friends. Therefore, most groups were fully engaged in the activities and quickly got into a party-like atmosphere, which lead to the fact that many laughs were rather expressive. Because they were not aware we were investigating laughter, there should be no element of laughing out of social desirability. For the same reason, there was no reason for the participants to produce posed laughter. Therefore, we consider our laughter as natural and spontaneous in nature.

After the five activities, participants filled out several questionnaires, were debriefed about the true purpose of the experiment, and received payment for participation.

#### Recordings

Recording took place in summer 2011 in a standard office room (7 × 4 m). The room was divided into two parts by heavy curtains so that the participants were on their own during the activities and the instructor and the recording equipment were not visible to them. In order to reduce the noise level and to get a good sound quality the room was lined with mineral wool walls covered in thick cotton fabric, carpets and heavy curtains. To limit the noise of the other participants, we build low divider walls from mineral wool covered with fabric. This proved effective to limit low volume noises such as rustling, breathing, and alike, while at the same time allowed participants to freely talk to each other. Participants were separated by these mineral wool walls during all activities except the tickling. Due to these walls the participants could not see each other during the video clips.

Participants were equipped with Shure WCE6 lavalier microphones (Countryman Associates Inc., Menlo Park (CA), USA) of the type omnidirectional characteristics, medium sensitivity, flat cap, without foam covering. In this configuration, the microphones have a highly linear (flat) response characteristic and do not run into saturation even during very loud laughs. Microphones were attached to a pre-amplifier of type Shure RK100PK. The RK100PK was connected to a Focusrite Saffire Pro 40 firewire audio interface (Focusrite Novation Inc, El Segundo (CA), USA). This audio interface digitized the microphone signal with a sampling frequency of 44.1 kHz and 32-bit resolution. The digitized signal was recorded using a standard laptop running Windows XP (SP3) and the software packages Abbleton Live Lite 8 Focusrite Novation Edition Version 8.0.9 (Ableton, Berlin, Germany) and Saffire Mix Control Version 2.2 (delivered with the Focusrite Saffire Pro 40). Microphones were attached to the participants ear and the amplification of each participant’s signal was levelled individually using the hardware dials on the Focusrite Saffire Pro 40 so that artificial coughs and loud “ahhh” sounds resulted in a medium amplitude, leaving enough headroom for very loud laughs.

#### Assessment of emotional experience

In order to identify the participants’ emotional experiences triggered by the video clips we used a questionnaire presented at the end of the session. The questionnaire contained one question for each of the 45 video clips used in the two sets. Each question consisted of a title (e.g., ‘Bride gets fit of laughter in front of altar’), a representative still image for each video clip, which made it easy for participants to remember the individual clips. Each question had a number of responses the participant could choose from. The response labels were joy (German: *Freude*), contempt/taunt (*Hohn*), fear (*Angst*), disgust (*Ekel*), fun (*Spaß*), schadenfreude (*Schadenfreude*), cute-emotion (*Süß finden*)^33^, embarrassment (*Peinlichkeit*), pity (*Mitleid*); as well as the option to list additional responses. Participants were asked “Can you remember some of the video clips? Which of the following emotion did you have during watching the video sequence?” and could tick as many emotions as they like. This procedure took account of the fact that a video might, for example, be perceived as funny by one participant and as schadenfreude by another. They were encouraged to skip questions in case they could not remember the video or the experienced emotion.

In total, emotional experiences could be assigned to 606 laughs elicited by funny video clips. During analysis of the post-questionnaire, it became evident that the response categories ‘fun’ and ‘joy’, which have been stated as being the main affective states experienced during laughter in former studies^17^, were used very frequently and often in combination with other categories. This resulted in the fact that only a third of the stimuli were characterised by a single emotion only, for example ‘schadenfreude’. The remainder of the stimulus set was mostly a mix of one emotion with either joy, or fun, or the combination of joy and fun. A smaller proportion were stimuli based on a mix of several emotions in addition to joy or fun (n = 141), for example joy and schadenfreude and pity (these ’multi-emotional stimuli’ were not part of the present study).

To extract a sufficiently large stimulus set, we applied the following rules: (a) The response category ‘fun’ was always ignored, so that a video rated as being ‘fun’ & ‘joy’ was considered as a ‘joy’ video. (b) The response category ‘joy’ was ignored in those cases where an additional category was chosen, so that a video rated as being joy and pity (or fun & joy & pity) was considered as a pity-video. As an example, a laughter sequence called a ‘joy’-laugh might be associated with videos rated as joy only, or as fun & joy. ‘Pity’ laughter might be associated with videos rated as pity only, as pity & joy, as pity & fun, or as pity & joy & fun (equivalent for schadenfreude, cute-emotion, embarrassment, taunt, fear, disgust). Note that this is a highly conservative approach: Suppose a laugher truly felt joy and pity and reported this in the questionnaire, and a listener classifies this laughter as joy. The listener was actually right in their judgement, but we would have regarded this as a wrong response (because a ‘joy-and-pity’ laughter would be reduced to a ‘pity’ laughter). We could be more lenient and consider both, ‘joy’ and ‘pity’ as a correct response, which would increase the recognition rates, and in the given example this would be justified. However, we suspect that joy is a default category participants may revert to in case they don’t know the answer. Because this would likely hold for both participant groups (the laughers and their responses in the post-questionnaire as well as the listeners when rating) such default answers may incorrectly inflate the recognition rates. We decided that it is preferable to be too conservative than too liberal. Laughter based on videos which were rated only as ‘fun’ (n = 80), taunt (n = 5), fear (n = 2), or disgust (n = 12), as well as multi-emotional stimuli (e.g., where schadenfreude and pity were reported) were not part of the present study.

#### Stimulus material

Sound recordings were digitized (sampling rate 44.1 kHz, 16 bit), cut in individual sequences and normalized. Sequences containing verbal material, other nonverbal expressions, background noise, being over-modulated, of short duration (less than three laughter syllables), or produced with a closed mouth were excluded.

Based on theoretical and pragmatic considerations, in a second step six laughter categories were selected: Due to its special evolutionary role and potential link to animal vocalisations we included (1) laughter emitted while being tickled. As a prototypical positive laughter (bright side), laughter evoked by watching video clips during which the sender reported to feel (2) joy was included. To reflect the dark side of laughter, we chose laughter evoked by watching video clips during which the sender reported to feel (3) schadenfreude. In addition, in a more explorative style, laughter evoked by watching video clips during which the sender reported to feel (4) pity, (5) embarrassment, and (6) cute-emotion have been included. Note that it is not our intention to propose a taxonomy of laughter (sub-)types.

Testing all 381 laughter sequences in one single experiment would have been too long and demanding for participants, so that we split the experiment into three different ‘runs’ or ‘waves’ of data collection (see Table 1). Stimuli were assigned pseudo-randomly to each run and conducted with different participant samples. In all runs, four answer labels by which participants had to classify the laughter were used: joy, tickling, and schadenfreude were always used, plus a variable fourth category (pity in run 1; embarrassment in run 2, and cute-emotion in run 3).

**Table 1:** Information about the senders, the stimulus set derived from those senders and presented to the receivers, and the receivers for Experiment 1.

	Run 1	Run 2	Run 3	Total
**Senders**	**26**	**27**	**24**	51*
Male	10	10	14	23*
Female	16	17	10	28*
**Stimuli**	**141**	**104**	**136**	**381**
Joy	46	29	34	109
Tickle	32	32	36	100
Schadenfreude	30	24	31	85
Pity	33			33
Embarrassment		19		19
Cute-emotion			35	35
**Receivers**	**51**	**42**	**66**	**159**
Male	29	23	33	85
Female	22	19	33	74

Because there were too many laughter stimuli to be tested in one single experiment, the experiment was split into three ‘runs’ or ‘waves’ of data collection. Each run was based on different stimuli and participants. *The set of senders partially overlapped for the three runs, resulting in a total which is different to the sum across the runs.

If a sender produced a high amount of laughter of a certain label (e.g., joy), laughter sequences were drawn randomly from that label from that sender. This reduced the original stimulus set from 830 to 381 laughter sequences (23 male and 28 female senders, mean age 23.9 years, duration range 0.38—8.29 s).

For joy, tickling, and schadenfreude laughter the stimuli were pseudo-randomly assigned to each run, balancing for a roughly equal distribution of laughter type, sender sex, and sender identity (see also Table 1). For pity, embarrassment, and cute-emotion, the total number of stimuli (19–35) roughly equalled the number of joy, tickling, and schadenfreude stimuli per run (24–46). Thus, by presenting only one of those categories per run we were able to keep the number of stimuli per category roughly balanced.

#### Procedure

To determine the required sample size for the key analysis (one-sample t-tests to test for classification accuracy above chance level), we conducted a power analysis (G*Power 3.1). Based on the findings of Szameitat et al.^7^, who found a very large effect for posed laughter, we assumed a medium effect for spontaneous laughter. To identify a medium effect (Cohen’s d = 0.5) with an alpha-error of 0.05 and a power of 0.9, a minimum sample size of 44 participants is required. Due to practicalities of participant recruitment, we sometimes recruited more than the required numbers (see Table 1, Receivers per Run).

For emotional classification, 159 native German participants (85 men, 74 women; mean age 23.3 years, see Tab. 1) classified acoustical laughter sequences into the four categories. For this, four answer boxes were presented horizontally on the screen. Participants were asked to first listen to the full laughter sequence before giving their answer. They were able to listen to the same sequence as many times as they wished before giving an answer. Once an answer box has been selected, the next laughter was played. The stimuli within a run were presented in three blocks, and participants were able to have a break between each block. Participants judged between 104 and 141 laughter stimuli per run (see Table 1 for details).

All experiments were run online using PsyToolkit^34,35^, except for the dominance study in Experiment 2, which took place in a lab of the University of Tübingen, running on a standard computer (using Presentation, Neurobehavioural Systems). Participants used headphones, and practiced for 10–12 sequences prior to the experiment. There was no time pressure during the experiment, and the average total duration was about 25 min.

#### Statistics

For emotional classification, hit rates were calculated and compared to the guessing probability of 25% using one-sample t-tests, Bonferroni corrected for multiple comparisons. Furthermore, Wagner’s unbiased hit rate for correct classification (H_u_)^36^ and Wagner’s proportion correct (p_c_) were both calculated for each participant and laughter type individually, and compared to each other in paired-sample t-tests. The comparison of H_u_ with p_c_ accounts for false alarms, uneven stimulus distributions, and response biases^36^. Because hit rates and H_u_ are bounded between 0 and 1, hit rate analyses were confirmed using bootstrapping methods, and for the H_u_ analyses the calculated scores for H_u_ and p_c_ were first arcsine-transformed^36^.

### Results 1

#### Laughter type classification

**Figure 1:**
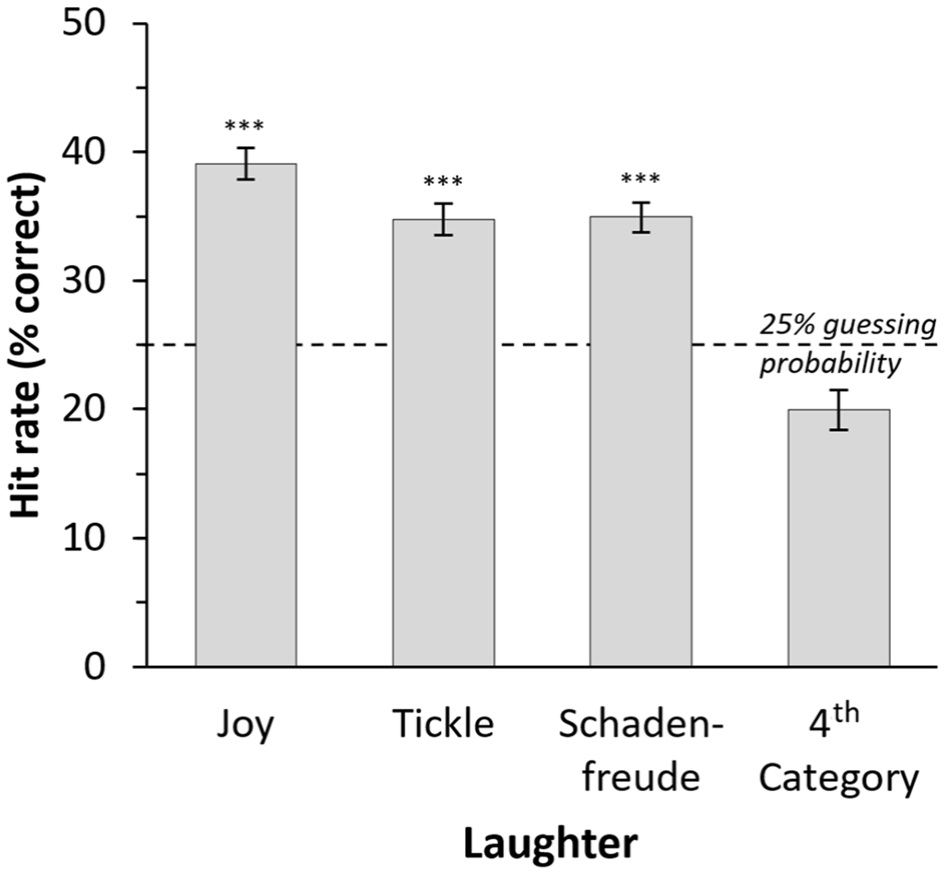
Classification results for Experiment 1. Data combined for all three runs (total N = 159; total number of stimuli = 381). In all runs, Joy, Tickle, and Schadenfreude laughter were presented, plus one additional fourth category (Pity in run 1, Embarrassment in run 2, and Cute-emotion in run 3). Data for the variable fourth category are averaged (see Results for details). ***Hit rate is significantly (p < .001; one-sample t-tests. Bonferroni corrected) higher than the guessing probability of 25% (confirmed by calculation of the unbiased hit rate H_u_^36^, see Results). Error bars denote standard error of the mean (SEM).

When data of all three runs in Experiment 1 were combined, joy, tickling, and schadenfreude laughter were correctly classified significantly above the chance level of 25% (Fig. 1; one sample t-tests vs. 25%; Bonferroni corrected for 6 comparisons; Joy: 39.09%, standard deviation (s.d.) 15.7%, 95% confidence interval (CI) 36.7–41.5%, t(158) = 11.284, p < 0.001, Cohen’s d = 0.898; Tickling: 34.76%, s.d. 15.36%, 95%-CI 32.4–37.1%, t(158) = 8.023, p < 0.001, Cohen’s d = 0.635; Schadenfreude: 34.93%, s.d. 14.45%, 95%-CI 23.7–37.2%, t(158) = 8.65, p < 0.001, Cohen’s d = 0.650). These analyses were confirmed using bootstrapping methods, which revealed virtually identical means and statistical significances. Statistical analyses for these combined data using Wagner’s *H*_*u*_^36^ confirmed these results (paired sample t-tests of *H*_*u*_ vs. the individually determined chance proportion *p*_*c*_; Bonferroni corrected for 6 comparisons; all ts(158) = 4.733–26.128, all ps < 0.001, all Cohen’s d 0.375–2.072). The variable fourth categories, i.e., pity (run 1), embarrassment (run 2), and cute-emotion (run 3), could not be correctly classified. Classification accuracies were in fact below the chance level of 25% in each individual run (Fig. 1, pity in run 1: 18.15%, s.d. 11.78%, 95%-CI 14.9–21.4%, t(50) = 4.288, p < 0.001, Cohen’s d = −0.577; embarrassment in run 2: 21.3%, s.d. 11.02%, 95%-CI 18–24.6%, t(41) = 2.174, p < 0.05, Cohen’s d = −0.336; cute-emotion in run 3: 20.35%, s.d. 10.48%, 95%-CI 17.8–22.9%, t(65) = 3.593, p < 0.001, Cohen’s d = −0.444; all *p*s uncorrected).

When the three runs of Experiment 1 were analysed separately, results were in line with the above findings from the combined analysis (see Tab. 2; hit rates compared to guessing probability, all ts = 5.542–9.162, dfs 41–65, all ps < 0.001, Bonferroni corrected for 4 comparisons), except for tickling laughter in run 1 (t(50) = 2.087, p = 0.042 uncorrected, n.s. after Bonferroni correction) and schadenfreude in run 3 (t(65) = 1.944, p = 0.056 uncorrected). Bootstrapping confirmed those analyses of hit rates. Again, for joy, tickling, and schadenfreude H_u_ overall confirmed the classification results when the runs were analysed separately (H_u_ vs p_c_; all p < 0.01 Bonferroni corrected for 4 comparisons; all ts = 3.119–19.236; dfs = 41–65), except for joy in run 3 (t(65) = 0.550, p = 0.584 uncorrected).

**Table 2:** Classification results for Experiment 1, separate for each run and across all runs (Overall).

	**Joy**	**Tickling**	**Schadenfreude**	**Pity**	**Embarrassment**	**Cute-emotion**
Run 1	44%***	30%^*ns1*^	42%***	18%**	–	–
Run 2	41%***	36%***	37%***	–	21%^*ns*^	–
Run 3	34%***	37%***	28%^*ns2*^	–	–	20%**
Overall	39%***	35%***	35%***	–	–	–

Shown are the hit rates (% correct) and their statistical significance (one-sample t-tests vs 25% guessing probability; Bonferroni corrected; *** p < .001; ** p < .01; ^ns1^ p = .042 and ^ns2^ p = .055 before Bonferroni correction; ^ns^ non-significant). – Laughter of this affective state was not presented in that experimental run.

The results show that receivers are able to decode the affective states joy, tickling, and schadenfreude expressed in laughter. The classification rates for pity, embarrassment, and cute-emotion were not statistically significant above chance level, which is why we focus the remaining analyses on the three categories of joy, tickling, and schadenfreude.

The confusion matrices show in more detail how the different stimuli were classified into the different categories (Tab. 3). Because a lot of stimuli carry multiple affective connotations (i.e., participants ticked more than one answer when rating the video clips, see methods section for more detail), we report confusion matrices based only on ‘single-category’ stimuli, i.e. where senders reported having felt only one single emotion. This reduced the number of responses from 20,493 to 10,908. The confusion matrices for runs 1–3 show that for each given stimulus category, the according response category was the most frequently chosen response, with the only exception of tickling in run 1, and leaving the varying fourth categories aside.

**Table 3:** Confusion matrices for the three runs in Experiment 1.

Stimulus	Response (%)			
Run 1	*Joy*	*Tickle*	*Schadenfreude*	*Pity*
*Joy*	43***	7	31**	19
*Tickle*	40***	30*	23	8
*Schadenfreude*	29	4	49***	18
*Pity*	33**	13	40***	**14**
Run 2	*Joy*	*Tickle*	*Schadenfreude*	*Embarrassment*
*Joy*	42***	9	32***	17
*Tickle*	30*	36***	21	13
*Schadenfreude*	26	9	53***	12
*Embarrassment*	36**	9	29	**27**
Run 3	*Joy*	*Tickle*	*Schadenfreude*	*Cute*
*Joy*	38***	11	30	21
*Tickle*	34***	37***	18	10
*Schadenfreude*	29*	19	30***	22
*Cute*	29**	13	34**	**23**

Data in bold represent correct classification. Asterisks denote significant recognition above 25% guessing probability (* p < .05; ** p < .01; *** p < .001; one-sample t-tests vs 25%).

To identify potential confusions, we tested whether other categories beside the correct target category also showed significant hit rates above the guessing probability of 25%. This revealed that joy was often confused with schadenfreude (significant in runs 1 and 2, not significant in run 3), but never significantly with tickle or the fourth category. Tickle was frequently confused with joy, but never statistically significant with schadenfreude or the fourth category. Schadenfreude was rarely confused with any other category, except with joy in run 3. Regarding the varying fourth categories, pity (run 1) was confused with joy and schadenfreude but not with tickling, embarrassment (run 2) was confused with joy but not with tickling or schadenfreude, and cute emotion (run 3) with joy and schadenfreude, but not with tickling.

### Discussion 1

The present results revealed that receivers were able to classify spontaneous joy, schadenfreude, and tickling laughter above chance level. Therefore, our findings demonstrate that laughter produced in different affective states of the sender can be discriminated by the receiver solely based on the acoustical signal without any further information about the situational context of the sender.

Comparing the present results with our previous findings on laughter produced by professional actors^7^, we found many similarities with respect to the general patterns in recognition rates and confusion matrices. For example, in both datasets joy, tickling, and schadenfreude laughter could be categorised above chance, whereby the classification rates in spontaneous laughter were slightly lower (spontaneous compared to acted laughter: Joy: 39% vs. 44%, Tickling 35% vs. 45%, Schadenfreude 35% vs. 37%). This is consistent with other research comparing posed with spontaneous acoustic vocalisations (see^37^ for emotional speech prosody; but^38^ for mixed results), and may be caused by actors over-emphasising emotion expression^24^, or the use of auto-induction technique for the recording of posed stimuli^7^, for which actors have been asked to get themselves into the emotional state as intensely as possible first, and then to laugh out freely. This procedure might have resulted in the actors expressing the emotions much stronger than felt on average during everyday social interaction.

Further similarities between the posed laughter^7^ and the current study could be found with regards to the confusion matrices. In detail, they showed that certain laughter types were more often confused with one type of laughter, but less with others, emphasising that not all laughter sounds alike. In both studies, schadenfreude laughter tended to be confused with joy (and vice versa), but less with tickling, which might be due to the fact that schadenfreude (i.e., experiencing joy in response to a mishap) carries the affective state of joy as well. Also, in both studies tickling was often confused with joy (in the post-questionnaire, 93% of ticklees reported feeling joy during tickling), but curiously this was not reciprocal, i.e. joy laughter was rarely confused with tickling, ruling out that these two laughter types simply sound very similar and cannot be discriminated. While overall the confusion patterns were rather similar across both studies, we also found some differences. For example, for posed laughter tickling was not only confused with joy, but equally often with schadenfreude, while tickling was confused 30–50% less often with schadenfreude than joy in the current study. The reason for this difference is unclear, but possibly tickling laughter can be less well portrayed than other laughter types.

Cute-emotion, embarrassment, and pity were not classified above chance level. There are several potential reasons for this finding. First, our particular set of video clips might have induced these affective states only weakly (we focussed on joy and schadenfreude and did not target clips which would primarily evoke other affective states), so that there was only a mild modulation of the laughter signal. Second, the classification approach might not be sensitive enough to reveal more subtle connotations. When participants hear a laughter sound which is perceived as joyful with an additional mild connotation of, e.g., cute-emotion, they are highly likely to select ‘joy’ instead of ‘cute-emotion’. Finally, the acoustical signal might simply not communicate these affective states, i.e., laughter might have evolved to express only certain affective states. A more fine-tuned methodological approach, such as allowing participants to tick more than one category, using gradual responses for each category instead of the binary answer format, or the inclusion of additional video materials eliciting additional emotions might answer these questions.

Note that we use the term ‘laughter type’ merely for pragmatic reasons to refer to the different categories in Experiment 1. It may be argued that laughter is a single vocalisation which does not have different categories, types, or sub-types. Instead, acoustical variations in this single vocalisation may be more gradual and could convey a range of potential meanings. This distinction is not relevant for the current paper, which aims to show that listeners are able to decode information about the affective context of the laugher.

It is noteworthy that we have followed a rather conservative approach. First, the stimulus set was unfiltered without any preselection. Second, laughter types actually showed conceptual overlap among each other, e.g., schadenfreude also carrying joy. Third, we removed this overlap by reducing, for example, a joy-and-schadenfreude stimulus to a schadenfreude-only stimulus, so that we most likely underestimated the true recognition rates (see Methods for details). Therefore, we consider the current findings as strong evidence that listeners can differentiate between different spontaneous laughter types.

At the same time, the findings also show the limitations of the chosen categorical approach. For example, it appears that the senders experience quite complex affective states, and more subtle connotations may be missed in a coarse four-category choice. To circumvent some of these limitations and to gain a more fine-grained insight into how the affective state might be communicated, in Experiment 2 laughter stimuli were rated according to four different emotional dimensions.

## Experiment 2

### Introduction 2

The second experiment aimed at gaining a deeper insight into how the affective state and the intentions towards the receiver^39^ might be communicated in laughter. For this, four different participant samples rated laughter sequences on a continuous scale according to the emotional dimensions of arousal, dominance, and valence. Regarding valence, it is noteworthy that the bright and the dark side of laughter potentially differ in their valence for sender and receiver. In particular, bright laughter based on positive emotions might be associated with positive valence for both, the sender and the receiver. On the other hand, while dark laughter might still be pleasant for the sender, e.g., because it is associated with a sense of superiority, it might be unpleasant for the receiver. To capture this potential difference, we assessed valence as two separate dimensions, the valence of the sender (i.e., in which affective state has the sender been when producing the laughter), and the receiver-directed valence, aimed at assessing the sender’s intentions towards the receiver (i.e. sender is eliciting a pleasant vs. unpleasant affective state in the receiver), resulting in four dimensions tested^7^. For ease of reading, we will include the receiver-directed valence in the term *emotional dimensions*, although it does not directly relate to the affective state of the sender.

### Methods 2

#### Stimulus material

For the evaluation of the emotional dimensions, a subset of stimuli from Experiment 1 was selected. In more detail, we chose those stimuli which were recognised in Experiment 1, i.e. which had a classification rate above 50%. The resulting stimulus set consisted of 121 laughter sequences (50 joy, 42 tickle, 29 schadenfreude, 1–4 per emotion and sender, 19 male and 20 female senders) with an average classification rate of 56,3% (tickle 52.8%, joy 54.5%, schadenfreude 63.4%).

#### Participants

A power analysis was conducted for the key analyses (paired sample t-tests to test for differences between laughter types) to determine the minimum sample size using G*Power 3.1. Szameitat et al.^7^ again reported large effect sizes for a comparable analysis using posed laughter, and we assumed a medium effect size for the current study with spontaneous laughter. To detect a medium effect with an alpha-error probability of 0.05 and a power of 0.9, at least 44 participants are required.

In total, 209 native German participants (85 men, mean age 23.5 years) took part in the emotional dimension study. Each emotional dimension was tested in a separate study. In detail, 51 participants took part in the Arousal study (mean age 22.8y., 23 female, 28 male), 63 participants took part in the Dominance study (mean age 24.7y., 32f., 31 m), 49 participants took part in the Sender’s Valence study (mean age 24.0y., 26f., 23 m), and 47 participants took part in the Receiver-directed valence study (mean age 21.8y., 24 m, 23f.). The dominance study has slightly more participants because it was also part of a further series of experiments (not reported here) requiring larger sample sizes.

#### Procedure

Laughter sequences were rated on a 4-point Likert scale (+ +| +| − | − −) according to one of four emotional dimensions: arousal (physically excited vs. calm), dominance (dominant vs. submissive), valence of the sender (sender being in a pleasant vs. unpleasant state), and receiver-directed valence (sender is pleasant vs. unpleasant towards the receiver, i.e. describing the sender’s intentions towards the receiver). The experiment was delivered in the same way as experiment 1.

Data for arousal, dominance, and receiver-directed valence were derived from independent samples, i.e. participants evaluated laughter only with respect to one emotional dimension. The sample for sender’s valence (N = 49) was mixed, consisting of 23 participants who were naïve to the experiment and 26 participants who already had participated in the arousal study. All participants were unaware of the three types of laughter included, and all but the latter 26 participants were unaware of the remaining three emotional dimensions tested.

#### Statistics

For emotional axes, the 4-point Likert scale (+ +| +| − | − −) was individually transformed for each laughter type and participant. In order to render a scale ranging from −100 to + 100, response frequencies were multiplied with a factor (1.5 for +  + , 0.5 for + , −0.5 for –, and −1.5 for − − ) and summed. This sum was divided by the highest possible sum (which depended on the number of stimuli in that category) and multiplied by 100.

### Results 2

**Figure 2:**
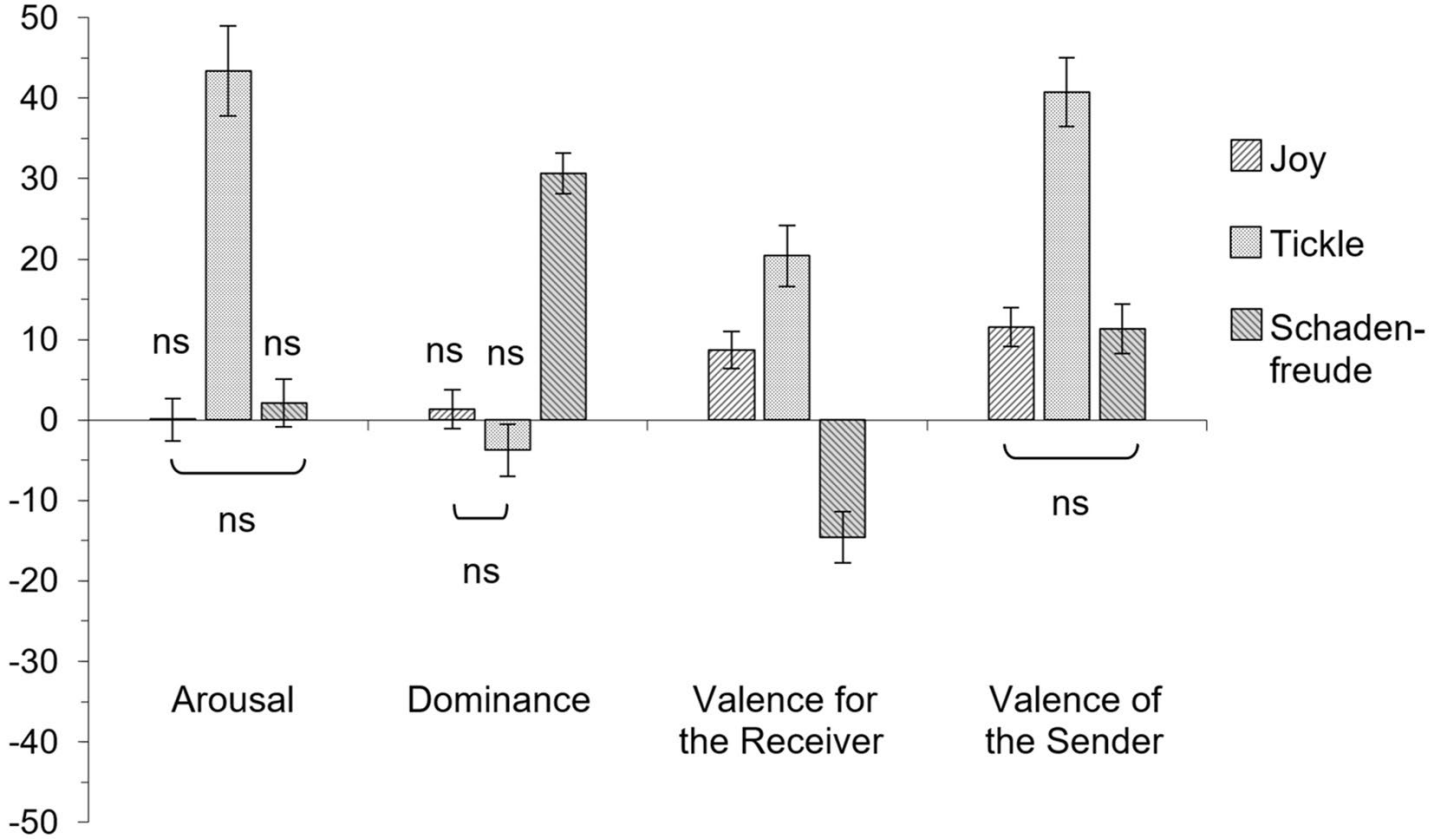
Rating results of Experiment 2. Three laughter types (Joy, Tickle, Schadenfreude) were rated on four emotional dimensions (Arousal, Dominance, Receiver-directed valence, and Valence of the sender) in independent studies. Answers from a 4-point Likert scale were transformed into a relative rating value ranging from −100 to + 100 (see Methods). All rating values differ significantly from zero and from each other within a particular emotional dimension (all ps < .05), except from nonsignificant ratings (ns). Error bars show SEM.

The evaluation of emotional dimensions shows that joy, schadenfreude, and tickling laughter differed with respect to all four emotional dimensions (Fig. 2). In detail, most of the laughter types differed from each other within each emotional dimension (Bonferroni-corrected paired-sample t tests, all *p*s < 0.05, except for joy vs. schadenfreude for arousal, joy vs. tickle for dominance, and joy vs. schadenfreude for sender’s valence). Furthermore, most of the rating values differed significantly from zero (Bonferroni-corrected one-sample t tests versus zero, all *p*s < 0.05, except for joy for arousal and dominance, schadenfreude for arousal, and tickle for dominance).

In detail, joy had both, a positive sender’s valence (mean 11.59, s.d. 16.95, 95%-CI 6.84–16.34) and receiver-directed valence (mean 8.71, s.d. 15.49, 95%-CI 4.28–13.14), while the arousal (mean 0.027, s.d. 18.66, 95%-CI −5.15–5.20) and dominance (mean 1.36, s.d. 19.31, 95%-CI −3.41–6.12) were neutral. Tickling laughter was characterized by a very high arousal (mean 43.39, s.d. 39.95, 95%-CI 32.31–54.46) and a very positive sender’s valence (mean 40.78, s.d. 29.87, 95%-CI 32.43–49.15). The receiver-directed valence was positive too (mean 20.40, s.d. 25.87, 95%-CI 7.40–13.00), while the dominance was neutral (mean −3.75, s.d. 20.06, 95%-CI 25.68–35.59). Schadenfreude laughter was highly dominant (mean 30.63, s.d. 20.06, 95%-CI 25.68–35.59) and was the only laughter type with a negative receiver-directed valence (mean −14.60, s.d. 21.68, 95%-CI −20.75 to −8.35). The sender’s valence was positive (mean 11.38, s.d. 21.53, 95%-CI 5.35–17.41), while the arousal was neutral (mean 2.11, s.d. 20.64, 95%-CI −3.61–7.84). Thus, schadenfreude laughter indeed showed the above-mentioned distinction between sender valence (positive) and receiver-directed valence (negative).

Statistical tests for cross-correlations revealed that some emotional dimensions correlated with each other (Pearson’s correlation coefficient, Bonferroni corrected for 6 comparisons). In detail, there were significant correlations for arousal and receiver-directed valence (r = 0.63, p < 0.001), for arousal and valence of the sender (r = 0.83, p < 0.001), and for receiver-directed valence and sender’s valence (r = 0.79, p < 0.001), while dominance did not correlate with any of the other emotional dimensions tested (with arousal: r = 0.03, ns; with sender’s valence: r = 0.16, p = 0.08, ns; with receiver directed valence: r = −0.17, ns).

### Discussion 2

The second experiment revealed that joy, tickling, and schadenfreude laughter showed distinct patterns along all four emotional dimensions, using stimuli with a recognition rate of at least 50% in Experiment 1. Therefore, the second experiment confirmed the results of the first experiment that the acoustical signal of spontaneous laughter can carry information about the affective state, and in addition showed that this information allows for an evaluation of emotional dimensions.

Our results support the common assumption that laughter has a bright side and generally is a positive signal^17^, at least for the sender: the valence of the sender was always attributed as being positive, even for schadenfreude. Note that this does not rule out that laughter with a negative sender’s valence also exists, e.g., laughter out of nervousness, sadness, or fear. While the existence of a dark side of laughter has long been speculated^7,16,18^, our results provide the first empirical evidence for the existence of spontaneously emitted acoustically distinct dark laughter: Schadenfreude laughter was perceived as negative for the receiver, and participants rated it as highly dominant. However, usually it is assumed that any negative effect is created solely by the situational context in which the laughter arises^17^, while the mere acoustical laughter sound has been postulated to be a truly positive signal^26^. While we agree that context can influence the interpretation of a laughter sound, the crucial finding of the present study is that context is not a necessary prerequisite for the occurrence of dark laughter.

In the present study schadenfreude laughter, as a form of dark laughter, was evoked by relatively harmless mishaps (such as somebody falling off a bike), but also some more serious ones (such as somebody stepping into numerous mouse traps as part of a prank). While it is socially accepted to laugh at such ‘bad luck’ of other people (e.g., expressed in dark humour), it is highly likely that laughter can get much darker in everyday life, for example when people play harmful pranks or when bullying others in school. It has been reported that people laughed in the aftermath of ’successful’, severely disturbing pranks^40^. Pranking has been associated with displaced aggression and the enjoyment of other’s experienced harm^41^. It might be speculated that such laughs are perceived as even more dominant and have a more negative receiver-directed valence.

When compared to our previous study based on posed laughter^7^, the current findings were again overall comparable. For example, in both studies tickling had the highest arousal, and schadenfreude was the only dominant laughter of the three laughter types. We also found differences, for example that schadenfreude had a significantly negative valence for the receiver in the current study, while it was rather neutral in our previous study. We believe that this difference is caused by the inclusion of taunt as a highly negative laughter in our previous study, which may have led to schadenfreude appearing less negative. Therefore, spontaneous and posed laughter appear highly similar in their emotional ratings, though possibly not identical.

Taken together, the second experiment demonstrated that spontaneous laughter can show variations along several emotional dimensions, and that these variations are not random as previously suggested^42^, but instead are linked to the sender’s physiological and affective state.

### General discussion

The present study investigated if natural spontaneous laughter can be differentiated according to the sender’s affective experience by naïve receivers based on the mere acoustical signal without any further contextual information. Experiment 1 showed that naïve listeners were able to classify joy, tickling, and schadenfreude laughter well above chance level. Experiment 2 revealed that joy, tickling, and schadenfreude laughter showed a unique pattern according to all four emotional dimensions tested, i.e. arousal, dominance, valence of the sender, and receiver-directed valence. The present findings show that laughter is not a uniform vocalisation with mere random variation in its signal, but instead that the variations in the acoustical signal can carry information about the affective state of the sender which can be decoded by the receiver.

It has been proposed that laughter functions via affect induction, i.e. spontaneous laughter heightens the receiver’s arousal and this heightened arousal, in turn, might elicit a learned affective response^43^. For example, one might experience a positive affect when being in a group of friends because their laughter is paired with previously established positive emotions^43^. In contrast, one might experience a negative affect when laughter is emitted in the underground/subway by a group of strangers, as the receiver might have negative presumptions about the situation^17^. If there is neither a positive predisposition nor any history of shared positive experiences, laughter is proposed to be less effective in inducing positive affect in the receivers^43^. The affect-induction hypothesis further states that acoustical variations in the laughter signal that may affect receivers in different ways, e.g. whether laughter is voiced or unvoiced, are not linked to the sender’s affective state^42^. The present findings show that the perception of laughter, in terms of classification and dimensional ratings, is linked to the sender’s affective state. For example, receiver-directed valence was linked to the affective content of the video material the sender observed while producing the laughter (i.e., positive receiver-directed valence for positive-natured videos, vs. negative receiver-directed valence for negative-natured videos). It is important to point out that all senders were in the same situational context while doing the experiment, i.e., together with a group of friends, and thus the situational context was constant for all laughter stimuli. Therefore, any approach suggesting that differences in laughter perception are solely caused by any form of context (social, situational, etc.) cannot explain the current data. Thus, while we do not rule out that affect induction is a potential mechanism, it cannot be the only mechanism in the communicative function of laughter.

A different approach to explain the communicative function of laughter is a representational approach, i.e. receivers decode information encoded in the laughter signal in order to gain knowledge about the sender’s feelings and motivations^44^. A strict representational approach would propose a near-perfect association between sent and perceived information, as it is usually the case for semantic content of speech. However, for nonverbal communication in general, the associations are less clear and therefore carry more probabilistic than definitive information. This can be seen in the current study, where the decoding accuracies of laughter were roughly comparable to those of other nonverbal communication channels, such as emotional prosody^45,46^. It is likely that in natural interactions this information is combined with further information sources, such as facial expressions, body language, and situational context, to shape the social interaction between the sender and receiver.

The question arises how could variations in laughter have evolved? Laughter has its phylogenetic and ontogenetic roots in being a play signal, and differences in laugh acoustics can already be found in non-human primates, for which laughs elicited by tickling (i.e., produced by the sender) show different acoustic properties than the laughs elicited by the laughter of others (the receiver “answers” with laughter, chorusing)^47^.

Then, with advances in human social interaction and increases in group size, laughter became independent from physical stimulation and started to serve the function of social grooming at a distance, in order to support affiliations in a peer group^15,18^. In line with this, the current results show that while tickling laughter is linked to a high sender’s arousal, joyful laughter has a low arousal, but still promotes positive valence for sender and receiver. A characteristic feature of both, tickling and joyful laughter, is that it is highly contagious and—already in pre-school children—often triggers a kind of chain reaction in a group^48^. Social grooming is the glue that binds social groups together, and laughter seems to be the perfect communication tool for reinforcing positive social relationships^17^.

In addition, group structure can be formed by segregating non-conforming individuals from the group^49^. In line with this, it has been shown that schadenfreude reactions about an out-group misfortune increase with affective ingroup identification^50^. Thus, from a certain age onwards, at latest in secondary school when social status and dominance become more important^51,52^, laughter can take on the form of laughing at others. The affective states investigated in our study map onto this theory, with Schadenfreude laughter being an excluding laughter, and tickling and joyous laughter being an including and bonding laughter. This supports the theory that spontaneous laughter can take on the form of a chorusing within a close social group, which transports a negative affect to the outsider. As an example for such a segregating laugh, laughter often arises when pupils laugh at a classmate in order to ridicule and belittle them as a form of bullying. Bullying is of value for the offender as it increases their level of popularity and social status within the social group^53,54^. Thus, we propose, that the function of dark laughter might not primarily be to communicate exclusion to the receiver (the stranger with the mishap), but first and foremost to establish the sender’s own inclusion and status in his own peer group. Schadenfreude laughter might offer the opportunity to enhance or restore a positive self-view when we compare ourselves to others. And while schadenfreude laughter might have its origin in harmless laughing about an incongruent situation^55^, it might represent the first step towards direct aggressive behaviour against out-group members^56^ being disguised as a playful signal. The present results show that such negative intentions for the receiver are directly encoded in the laughter signal highlighting the power of laughter in order to shape social relationships.

## Data Availability

Data have been made publicly available at Figshare and can be accessed at 10.17633/rd.brunel.15028296.
